# The Drug Aspect of Lesions of the Gums

**Published:** 1904-04

**Authors:** A. W. Harlan

**Affiliations:** Chicago, Illinois.


THE AMERICAN

### Journal of Dental Science.

### THE DRUG ASPECT OF LESIONS OF THE GUMS.

By A. W. HARLAN, A.M., M.D., D.D.S., Chicago, Illinois.

Read before the New Jersey State Dental Society, Asbury Park, July, 1903.

I have felt a great reluctance in coming before you with such a subject—not that too much has been written about it, but because what has been written falls too far short of the mark, to comprehend the intelligent and logical application of drugs to, and in the gums, and the administration of drugs internally, that will have an effect on the gum tissue and adjacent mucous surfaces. When we look at the gums as they surround the teeth and cover their bony sockets, we are led to think that the picture presented will always last; that the crescentic margins will never shrink, waste away nor become the seat of disease. So soon, however, as the loss of a tooth, the lodgment of a piece of wooden toothpick between the teeth, the passage of a file or disc between them, or the application of a clamp, separator, wedge or any other tool that will injure the gingival margin has begun the initial destruction we are confronted with a lesion that if neglected may become irreparable. Let the possessor of perfect gums neglect to clean the teeth for a few days or weeks, and there will be destruction of the gingival margin. Let him suffer from some malady that will confine him to his bed for a few weeks or months, and the gums will show the effects of such illness.

I need not dwell upon the many known causes of the beginning of destruction of the gums, for the most of them are known to you. An abscess, a badly fitting clasp or plate attached to the teeth, the bands and bars of regulating appliances or ligatures, all have their effect upon the gums, and many of them leave their mark there for you or me to remedy as best we can.

Specific diseases and bad nutrition are chargeable with some defects of gum tissues. Tobacco and salivary deposits make their impress on the gums, and mouth breathing has a large part in distorting the teeth and disarranging them in their sockets. Any affection of the mucous membrane of the nose, mouth or pharynx that is more than transitory, will affect the gums. Lack of personal care and cleanliness of the teeth is responsible for many of the defects of gum tissue, and the injudicious use of tooth picks and ligatures or rubber bands will do much to make permanent injuries to the gums. All foreign bodies between the teeth and under the free margins of the gums are injurious, such as insoluble particles of ingredients of dentifrices. All washes and lotions and pastes and salves, having free acids or corrosive acids, by rendering the teeth sensitive, injure the gums, because then they are not well cleaned.

You may guess that there are many other agencies that do harm in some way to the gums, but one which has not been dwelt upon by any writer during the past ten years is the growing habit of feeding children and adults with finely comminuted prepared breakfast and luncheon foods, many of which are predigested and placed before the sufferer in such a form that they do not require mastication at all! Why are we endowed with thirty two teeth if they are not to be used to bite, tear and chew food? The teeth need the exercise and the gums need the friction, and as we look at it now they get neither the one nor the other in this age of ready-to-eat foods, many of which are no doubt nutritious, but not needed by the great mass of men and women who must work and exercise other portions of the body. If we are to keep our teeth in number, and form, and health, they must do their share of the work of the body to justify their place as organs. Foods and foodstuffs should be cooked or prepared in such a manner that they require mastication, and when eaten raw one of the pleasures of life is lost if they are bolted without mastication. You must realize that the gums are a part of the human economy, and you may take lessons from the lower animals, who eat slowly and carefully to insure perfect digestion. In order to have good, satisfactory masticating teeth, the gums must receive friction from the food you eat or they should be rubbed in some manner to insure perfect circulation and health. Teeth without gums are denuded teeth, and in many cases they are useless teeth. If you would save your patient much distress keep his gums in order, or make him do it, or show him how to do it. The teeth need their natural contour; if this is lost the gums suffer. This contouring of teeth must be applied in the making of crowns and bridges in order to protect the gums. An irritated or inflamed gum from lack of contour of the teeth will remain so, and even degenerate if the contour is not restored. I look upon the proper care of the gums from the personal standpoint as of as much importance as the care of the teeth themselves.

A lesion of the gum may be local, a physical injury, or it may be pathologic. Many, if not all the injuries, will recover with little or no treatment, if they are superficial, or if the cause of the injury is removed quickly, as the cutting away of a protruding filling, the removal of any exciting cause of trouble between the teeth. A crown which is too long may cause serious trouble to the gums and sockets of teeth if not cut away, or even a large filling that does not occlude properly will cause much trouble; swelling of the gums, even death of the pulp has resulted from such cause. There is usually no drug aspect of the gums in purely local injuries, unless we include the application of an astringent, as zinc sulphate, lead water, boracic acid or one of the naphthol series in saturated solution. Most of the local hemorrhages are controlled by hot water, ferri-pyrine or adrenalin chlorid. Tannic acid, zinc acetate or alumnol could be used in such cases. When there is a suspicion of infection from a local injury 1 to 1000 corrosive sublimate, hot, 105 degrees F., or argyrol or mercurol will suffice. In the absence of all of the above, first cleanse the surface with hot salt water and paint with carbolic acid 95 per cent, or treat the surface with 25 per cent trichlor acetic acid. Give the patient fifteen grain doses of soziodol, or four grain doses of quinine bisulphate every hour in one ounce of spirits.

Any case of local infection of the gums or mucous membrane of the mouth must first be cleansed, second disinfected with local applications, and third internal administration of a drug that will combat sepsis—salol, iodine compounds, boric acid, mercury, the coal tar derivatives, such as the naphthol series. These cases generally are cared for by practitioners of medicine, but any dentist should know what to do and do it at once.

The lesions of the gums, having a distinct drug aspect, are those brought about through neglect, or by the presence of organisms, usually pus producing organisms. A lesion defined as accidental, local or pathologic will need to be treated as such. Some general diseases have their effect upon the gums, as scrofula, syphilis, ptomaine poisoning, continued fevers and the exanthemata. Filth, poor nutrition and faulty assimilation are chargeable with gum lesions. The drug aspect of general diseases is limited to the local use of sprays, washes and general cleansers. Boracid acid, peroxide of hydrogen, sublimale solutions, Beta, or hydronaphthol solutions, silver compounds, iodine, zinc, copper and strictly acid solutions, acetic, hydrochloric, tri-chloracetic, etc. The poorly nourished and those who do not assimilate need change of air, water and their general surroundings, as much as they need internal tonics; sometimes indeed they get well without anything more than sea air or mountain breezes, with a change in diet and water. On several occasions I have sent my patients to French Lick Springs or West Baden, Indiana, and after ten days or two weeks found them much benefited by the use of the waters. In prescribing waters I find that drinking them is absolutely essential, as no benefit comes from the bathing alone. All cold waters simply give a shock and tone to the skin, and the heated waters by raising the temperature assist in eliminating poisons. None of the waters are absorbed to a degree sufficient to be considered as medicinal.

Some very exellent ideas on water may be found in Baruch's classic and in T. Sollman's work on Pharmacology.

The lesions of the gums requiring drugs are those proceeding from the apices of roots, the sides of roots and the surfaces of the gums themselves. In the latter the treatment is very simple. If the gums are cleansed with peroxide of hydrogen and then painted with the compound tincture of iodine, two or three coats, so as to get a new surface, and the patient is fed on liquid or semi-liquid food for two or three days, the gums will become clean and free from redness, irritation or a tendency to bleed. If a discharge exudes from between the gum and the root of a tooth, usually there is some concretion on the root, which must be removed mechanically or surgically, and the pouch or pocket cleansed or burned with some drug, as acetic, sulphuric, lactic, tri-chloracetic or citric acid in solutions in water of from 10 to 50 per cent. If the cutting and gouging have been well done, and the teeth are firmly lashed to each other or held by bands, and the medicine well injected in sufficient strength, Nature will have an opportunity to discharge all effete matter.

If the lesion proceeds from the apex, it means a dead pulp, and anything from cinnamon water to 95 per cent carbolic acid could be used through the root to irrigate the fistula and the root may be filled with your favorite root-filling: wood, bone, silk, oxychloride, wax, gutta percha, cotton, silver, copper or gold wire, salol or chalk. Abscesses do not heal, to stay healed, unless the end of the root is sealed. Sometimes it is necessary to amputate the portion of root necrosed before the lesion will disappear.

I am a strong believer in keeping the gum margin intact, therefore I do not favor the indiscriminate use of wooden toothpicks, floss silk or rubber bands. The patient who expects to keep his gums in order must have his teeth well contoured; they must occlude well. All crowns or bridges should be contoured on the portions that are attached to roots of teeth. The gums must be scoured and washed and massaged daily to prevent tendencies to bleed. If the teeth are sensitive to the air or to touch, magnesia, chalk, soda or lime must be used to counteract this. If the gum has been denuded from the neck of a tooth and the patient is still young enough —say under forty—you can coax it to grow and recover the root in part by great care, and stimulating it gently with iodine and zinc iodide and 10 per cent resorcin painted on it at intervals of one to two weeks. This is very tedious and requires much patience; these remedies must be alternated and changed about every six weeks. When you are trying to build gum tissue the patient must use a Badger brush and a dentrifrice composed of one part white castile soap and nine parts of precipitated chalk. Occasionally I add 20 grains of powdered quillai saponaria to a 10-ounce prescription. This may be perfumed with any oil and colored with carmine or cochineal.

The gums always do well when they are properly rubbed or massaged with water about 53 to 60 degrees F.; warm water is relaxing. Dentrifrices are for the teeth, not the gums. Washes are for the gums, and the mucous membrane of the mouth. Washes are intended to be used as remedial agents and germ destroyers.

A very useful wash is composed of:

Hydronaphthol or Beta napthol. . ʒ i

Menthol . . . . . . . . . . . . . . . . . . . . . . . gr ij ss

Oil cinnamon or cassia a a . . . Min V

Oil wintergreen. . . . . . .

Glycerine a a . . . . . . . . . . . . . . . ℥ iij

Alcohol

Distilled water . . . . . . . . . . . . . . . ℥ x

℞. Sig. Use as a mouth wash, full strength.

Mouth washes are like the hairs of your head—numberless, but as they are used they do not accomplish very much. In order to be effective the mouth must be scrubbed first, and then rinsed with cool water, and the wash is then introduced in a small quantity, ½ teaspoonful; the gums are rescrubbed for from one to two minutes, and the effect of the wash left to be diluted with the saliva. Even with this care the organisms are not all destroyed as a rule.

Any treatise on the gums would be incomplete if it did not consider the possibility of the use of the Finsen ray for the arrest of pus production. I have for a few months been using this ray in a limited number of cases, and it appears to me that we will have less use for drugs in the so-called pyorrhea after a year or two, than we now have. There are still some difficulties to be overcome in the general use of the Finsen system, in its application to the gums, but they are not insurmountable. The bactericidal effect of the ultra-violet ray is produced in something like four to five seconds in a field, with full exposure, by a Bang lamp,* while the arc light exposure requires about five minutes to destroy a culture of the staphylococcus pyogenes aureus. A slight erythema lasting for some days may always be expected in the use of the ultra-violet—the invisible ray. If this is brought in contact with the hair follicle, the hair will drop out and the nails may become brittle.

In the treatment of the gums these accidents are not likely to occur.

Very shortly there will be on the market a sufficiently simple apparatus for gum treatment, which will make it possible for any one to use the ultra-violet or invisible ray, even in the treatment of other affections of the gums.

From many years of observation of the gums I am convinced that the instruction given by the dentist to the patient is not sufficiently clear or impressive to do the greatest good, therefore I appeal to you to give a demonstration every time you find any discharge from between the gum and the tooth to make it an object lesson. Cases of this kind may improve with drugs alone—but a certain amount of delicate and exact surgery is requisite to effect a cure which will not be permanent unless great care is exercised continuously by the patient.—*Items of Interest.*

*Turner Medical Electricity, 3d edition.

DISCUSSION.

Dr. R. M. Langer, East Orange:—The subject as presented by Dr. Harlan is practical, thorough, dental prophylactics—a subject which cannot be thrashed too hard or too often in my opinion. It is a strange fact—well, hardly strange, but a fact—that as civilization advances the dental organs seem to deteriorate. Whether good living necessarily means bad teeth, or whether in our age of effete civilization we are too lazy to get that which is so easily attained, I am not prepared to say, but certain it is that the higher up we go in the scale of civilized life, the more abundant are poor teeth and apparently the more common is the neglect on the part of the patient. Many seem to think that the dentist can do it all, and so we are obliged, whether we will or not, to undertake the task, and we can only, I am sure, hope to aid to the degree in which we succeed in educating our clientele to do the work for themselves instead of expecting us to do it.

The reference to breakfast foods is one of the indications. I heard an anecdote the other day to the effect that a gentleman said to a saw mill owner, "How is it that you are working now? I thought it was your dull season?" "Well," he said, "since they have started the breakfast food business, we are running the year around." The tendency of the age to live on predigested food is not only shown in poor teeth, but I think is equally shown in the anaemic children who are coming along to make the next generation. The only thing which is saving them from their own neglect is the popularity which outdoor exercises enjoy in this decade.

Just a word on the practical side of the question in regard to injury to the gums and teeth in band fitting. I read a paper recently in one of the journals which said that the only correctly fitted band around the neck of the teeth was the one which was driven home with a mallet. I have seen bands put on in that way that had their only claim to a fit is the "fit" that the patient had while the mallet was being applied! The operator who has had a few bands fitted in that way finds a way of fitting them perfectly without the mallet. With reference to placing ligatures near the gums, I would like, if it were possible, to ask how many men anesthetize the gingival border before they place a band or tie a ligature? How many men take the trouble to apply a little cocaine or camphophenique or some equally efficacious remedy?

Some years ago the S. S. White Co. introduced a dental chewing gum. I presume it is safe to say that most of you do not approve of chewing gum—for the other fellow—yet there was a principle underlying that very remedy, if we may so call it, which is wise to consider. I know very well that if you recommended parents to have their children chew gum so often every day they would probably call in another dentist, but at the same time the massage produced by the act of chewing gum is of inestimable value sometimes.

That we hope great things from the Finsen ray is unquestionably true. The fact of the matter is that this is an age of electricity, and by and by, if we are not careful, our vocation may be gone, because they will just put the man who needs treatment in an electric cabinet, touch the right button and turn him out cured!

---

Dr. Louis C. Leroy, of New York: One of the opening paragraphs of Dr. Harlan's paper reads "That a lesion (of the gums) if neglected may become irreparable." I would define that somewhat closer by saying that all injuries to these tissues, *if* involving the peridental membrane, become irreparable. They may respond temporarily to stimuli, but the scar remains which in after years breaks down as a poorly resisting tissue, and that insidious malady, gingivitis, present itself.

The paper quotes a number of irritants which operate as contributing causes of lesions. A few others might be added, such as necrotic lesions, irritation from poisons, bristles from tooth brushes, or small fish bones sometimes create disastrous irritations.

The floss silk, too, can do much damage through improper use or too rigorous use. If it is used carefully, it is practically indispensable, especially in mouths where the teeth are irregular or the jaws deformed. If the patient must use a tooth pick the quill is the only thing. The reasons are obvious.

Just a few words about massage. In conditions of anemia, and in fact in many diseases of a different character, after the usual brushing of the mucous membrane of all surfaces of the mouth, massage that tissue, particularly over the aveolar processes, by grasping it between the thumb and forefinger and exerting a vigorous pinching influence, dragging the tissues from the apical part of the teeth to the cutting edges, exerting a mild twisting motion when the crowns of the teeth are in the fingers. This done two or three times daily will accomplish excellent results. The action of the epidermis on the mucous surfaces has a cleansing action and stimulates the mucous follicles in a way that the tooth brush or other instrument does not. I have in mind cases where the gums have been diseased and the teeth loose from disuse that have become quite firm again under this treatment.

Dr. Harlan referred to prepared foods. That has been a recognized evil for some time past which together with the general malady human beings suffer from—inactivity and sedentary habits—results in general impaired functions. The child, in its endeavor to relieve the distress of hunger, accepts the palatable diet which is served to it by the parent, ignorant of the simple requirements for proper functional activity.

Among the more refined, especially in cities, another insult to injury is heaped on suffering childhood by practically depriving it of muscular activity, which is necessary for proper functional activity of all organs of the body, particularly the digestive. Suppression of these functions at this period of the child's life results in chronic atrophic conditions of the growing organs which time and change to activity can never correct. For exercise the child is allowed to walk up and down the pavement, hitched to a nurse or, worse still, seated beside her for fear she might soil the spotless garment or person. Is it any wonder that lesions of the gums and teeth and stomach and the whole alimentary tract are prevalent in the growing generation and that malnutrition asserts itself early in life? Adults ignorantly suffer in somewhat the same way.

What constitutes exercise? Surely not walking as generally indulged in, but good brisk activity that will cause, above other things, free flow of perspiration. That is a requisite to eliminate the waste products stored in the circulatory system and which cannot wholly be gotten rid of by any other means. A few minutes of judicious exercise should not exhaust any individual, but on the contrary, stimulate. Exhaustion is dangerous. My reason for placing so much stress upon exercise is because of personal benefits and injuries and the observation of others, in and out of practice, and of athletes who have been in training, gone out and returned to activity again.

The judicious, systematic sweat—pardon the inelegancy—with proper laving after will do much to correct the existing evils of gum lesions and many others, foreign to detistry, and even solve the dietetic problem, for the whole system will crave those things which will regulate proper functional activity best. At present, the predigested food is a result of conditions rather than accountable for them.

Then, too, the irrational drinking, or not drinking enough water, for purposes of health as well as the infrequent use of it for bathing, requires careful consideration.

As to the drug aspect of Dr. Harlan's paper, I have not much to say. His medical knowledge is based upon such positive rules that any departure therefrom is simply judgment or idiosyncratic preference of a drug and its intelligent administration.

I am very much pleased with Dr. Harlan's reference to the Finsen ray. How we will welcome the ultra-violet principle with all its possibilities.

If Dr. Harlan will inform us where that practical Finsen principle may be obtained I feel confident the results pro and con of the experiments that will follow will monopolize much of the 1904 meetings of all societies.

Dr. Wilbur Daly, of New York: Dr. Harlin spoke of the use of bichloride of soda. Ordinary mouth washes are of the ordinary myrrh type and the boracic acid preparations. This acid has a tendency to be a coagulant and works on the cervical margin by contraction and tends to denude the dentine of gum tissue and also affords a place of deposit of bacilli. Bicarbonate of soda dissolves certain mucous secretions on the surface of the teeth, especially where the mucous in the saliva is in larger predominance than ordinary in healthy saliva. The saliva is one of the first things that indicates malnutrition; its chemical conditions change and the waste products of the body accumulate in it. The bicarbonate of soda will wash the surface of the teeth and remove the film which accumulates and thus prevent the accretions of tartar and also prevent little gum lesions.

---

Over Medication.—In reference to over-medication in the treatment of pyorrhea, you cannot over-medicate the part, for the reason that the saliva carries the medicine away as fast as you put it in there. If you wish to rely upon any drug for action in a pus pocket, or on the side of the root, let the drug be in the form of a powder, not in a solution.—*Dr. W. H. G. Logan, Review.*

